# Isolation and kinetic characterisation of hydrophobically distinct populations of form I Rubisco

**DOI:** 10.1186/1746-4811-10-17

**Published:** 2014-06-12

**Authors:** Kerry O’Donnelly, Guangyuan Zhao, Priya Patel, M Salman Butt, Lok Hang Mak, Simon Kretschmer, Rudiger Woscholski, Laura M C Barter

**Affiliations:** 1 Institute of Chemical Biology, Department of Chemistry, Imperial College, Flowers Building, South Kensington Campus, Exhibition Road, London SW7 2AZ, UK; 2 Department of Chemistry, Imperial College, South Kensington Campus, Exhibition Road, London SW7 2AZ, UK

**Keywords:** *Brassica oleracea*, Hydrophobic interaction chromatography, Purification, Rubisco, *Spinacia oleracea*

## Abstract

**Background:**

Rubisco (Ribulose-1,5-bisphosphate carboxylase/oxygenase) is a Calvin Cycle enzyme involved in CO_2_ assimilation. It is thought to be a major cause of photosynthetic inefficiency, suffering from both a slow catalytic rate and lack of specificity due to a competing reaction with oxygen. Revealing and understanding the engineering rules that dictate Rubisco’s activity could have a significant impact on photosynthetic efficiency and crop yield.

**Results:**

This paper describes the purification and characterisation of a number of hydrophobically distinct populations of Rubisco from both *Spinacia oleracea* and *Brassica oleracea* extracts. The populations were obtained using a novel and rapid purification protocol that employs hydrophobic interaction chromatography (HIC) as a form I Rubisco enrichment procedure, resulting in distinct Rubisco populations of expected enzymatic activities, high purities and integrity.

**Conclusions:**

We demonstrate here that HIC can be employed to isolate form I Rubisco with purities and activities comparable to those obtained via ion exchange chromatography (IEC). Interestingly, and in contrast to other published purification methods, HIC resulted in the isolation of a number of hydrophobically distinct Rubisco populations. Our findings reveal a so far unaccounted diversity in the hydrophobic properties within form 1 Rubisco. By employing HIC to isolate and characterise *Spinacia oleracea* and *Brassica oleracea*, we show that the presence of these distinct Rubisco populations is not species specific, and we report for the first time the kinetic properties of Rubisco from *Brassica oleracea* extracts. These observations may aid future studies concerning Rubisco’s structural and functional properties.

## Background

Rubisco (Ribulose-1,5-bisphosphate carboxylase/oxygenase) is the CO_2_-fixing enzyme in the Calvin cycle, the primary pathway of carbon assimilation in photosynthetic organisms. It catalyses the reaction between Ribulose-1,5-bisphosphate (RuBP) and CO_2_ to produce two molecules of 3-phosphoglycerate [1,2]. It is reported that Rubisco is responsible for a net fixation of 10^11^ tons of CO_2_ from the atmosphere to the biosphere per year [3]. Despite its importance, Rubisco is a remarkably inefficient enzyme, suffering from both poor specificity and a slow catalytic rate. It is not surprising that this enzyme has generated much interest, as it is suggested to be one of the major bottlenecks limiting maximum photosynthetic efficiency [4]. The efficiency of carbon fixation is reduced by side reactions, the most notable being photorespiration, where Rubisco fixates O_2_ instead of CO_2_. Photorespiration imposes a significant metabolic constraint, lowering the efficiency of carbon fixation by up to 25-50% and constantly draining the pool of available RuBP [5,6]. Rubisco is the most abundant protein found on the planet, making up approximately 50% of the total leaf protein [7]. This however comes at a cost in terms of the plant’s nitrogen requirements [1].

Rubisco is a multimeric enzyme comprising varying numbers of the large (50–55 kDa) and small subunits (12–18 kDa), which in higher plants and green algae are encoded by the chloroplast genome and the nuclear genome respectively [2,8]. There are a number of different forms of Rubisco (forms I, II and III, as well as the more diverse Rubisco-like proteins (RLPs) called form IV), reviewed by Tabita *et al*. [9]. Form I Rubisco is the most common, found in higher plants, cyanobacteria and eukaryotic algae [2], and its structure has been solved in many species, but first reports were for tobacco [10] and spinach [11]. It consists of eight large and eight small subunits in a hexadecameric structure, forming a barrel of four large subunit dimers arranged around a four-fold axis of symmetry, capped by four small subunits at each end [8,12]. The large subunits play a catalytic role, however the precise role of the small subunit is not clear, as it is not essential for catalysis [13,14]. Interestingly hybrid enzymes which contain large subunits and small subunits from different species have been reported to show differences in their stability and specificity [15-17].

There is a lack of understanding of the interactions and engineering rules that control and regulate Rubisco’s activity, although this is critical if we are to increase photosynthetic efficiency. A prerequisite for the characterisation of the structure and function of Rubisco, along with investigations into its interactome, is the need for rapid methods that can isolate Rubisco with high purity. Purification methods in the past have taken advantage of the enzyme’s negative net charge under physiological pH, which make it suitable for anion exchange chromatography [18-21], as well as the relatively high molecular weight of the hexadecameric holoenzyme, which made it an ideal candidate for size exclusion chromatography, or sucrose gradient centrifugation [22-25]. In addition, differential ammonium sulphate ((NH_4_)_2_SO_4_) precipitation has been employed prior to the ion exchange chromatography (IEC) [19,24]. A number of the reported high purity Rubisco isolation protocols are lengthy, due to the need for dialysis or ultra-centrifugation to remove sucrose prior to assaying the sample, which leads to an increased risk of endoproteolytic activity on the homogenized leaf tissue [26]. Given the wide use and effectiveness of (NH_4_)_2_SO_4_ precipitation it is surprising that hydrophobic interaction chromatography (HIC), which is based on similar separation principles, has only been explored once as a purification technique for form III Rubisco from recombinant *A. fulgidu*, but has yet to be employed for form I Rubisco [27].

The potential of HIC as an alternative to IEC to obtain form I Rubisco with high purity was explored, since it is ideally suited as the proceeding step to the commonly employed (NH_4_)_2_SO_4_ precipitation in Rubisco purification protocols [28]. Employing (NH_4_)_2_SO_4_ in purification protocols may have the added benefit of removing endogenous inhibitors, since the presence of sulphate ions in Rubisco extraction buffers has been shown to remove and prevent the binding of inhibitors such as 2 carboxy-D-arabinitol 1-phosphate (CA1P) [29,30]. (NH_4_)_2_SO_4_ precipitation results in samples with high salt content, which require dialysis, desalting or dilution prior to the subsequent IEC purification step. In contrast, HIC can exploit the high salt content of the (NH_4_)_2_SO_4_ precipitate, therefore avoiding any further dilution or desalting steps. HIC, like IEC, offers the added advantage of being able to bind proteins from large sample volumes, which is in contrast to gel filtration chromatography and sucrose gradient centrifugation, often employed in addition to IEC [19,24].

We demonstrate here a rapid purification method that, for the first time, is able to reveal hydrophobically distinct Rubisco populations. Our results confirm that the HIC protocol, detailed here, can be employed to obtain highly purified Rubisco from *Spinacia oleracea* (*S. oleracea*, *Spinach*), with kinetic properties in agreement with literature values from samples subjected to commonly employed purification protocols [20,31].

Once verified by this comparison, the HIC method was utilised to purify Rubisco from another species, the so far uncharacterised enzyme from *Brassica oleracea* (*B. oleracea*, *Cabbage*), revealing for the first time the kinetic properties of Rubisco from this species.

## Results and discussion

**Figure 1:**
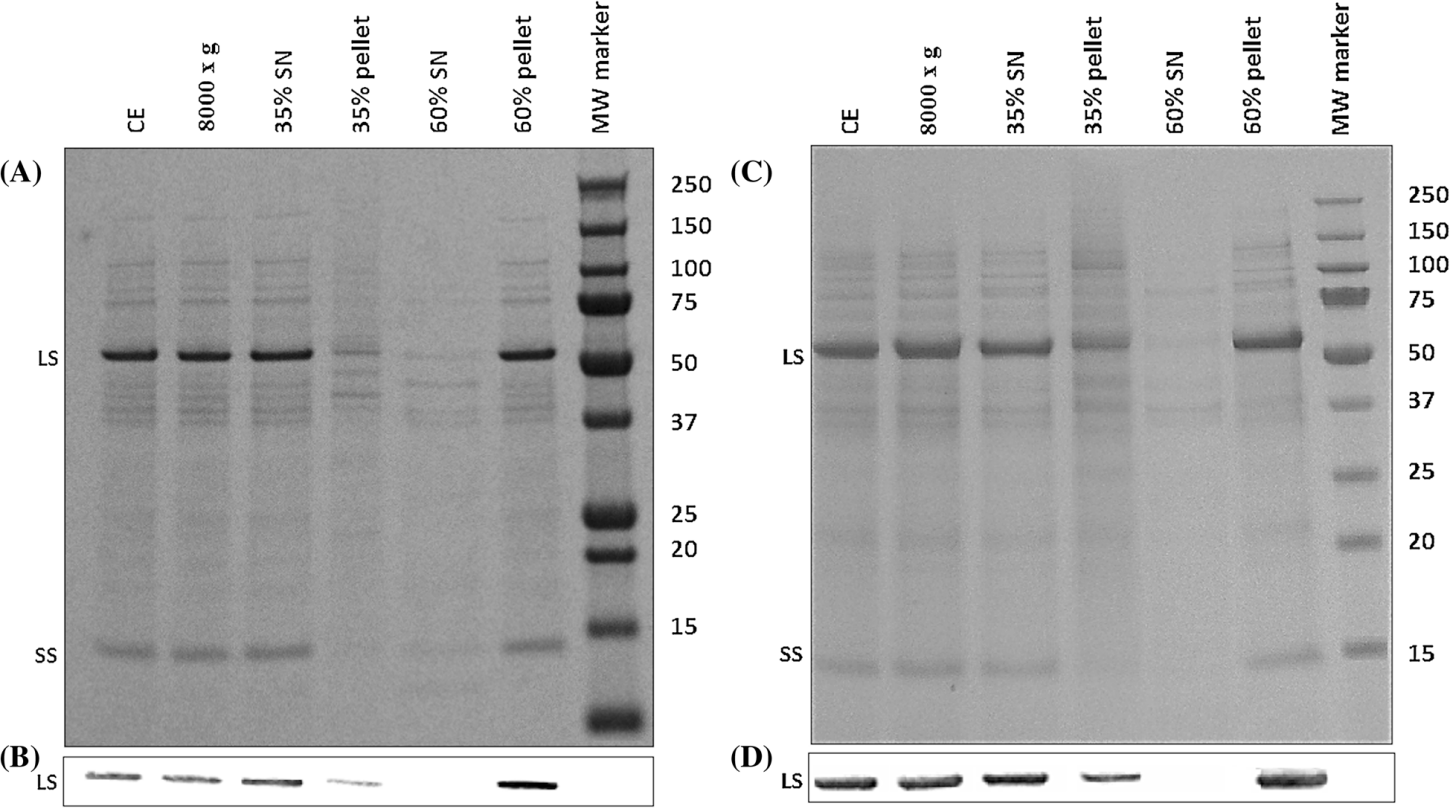
**SDS-PAGE gels and western blots showing protein content in ammonium sulphate fractions from *S. oleracea *and *B. oleracea. *(A)** SDS-PAGE gel showing protein content in ammonium sulphate fractions from *S. oleracea*; Extracts were subjected to ammonium sulphate precipitation as described in Methods. Samples were separated by SDS-PAGE and subsequently stained for protein. The position of Rubisco’s large (LS) and small (SS) subunits are indicated in the figure. 0.4 μg of sample was added per lane. **(B)** Western blot showing Rubisco content in ammonium sulphate fractions from *S. oleracea*; The relative intensities of the LS were calculated to be 0.85 ± 0.04 (CE), 0.89 ± 0.01(8000 × g), 0.92 ± 0.02 (35% SN), 0.55 ± 0.15 (35% pell) and 1 (60% pell). **(C)** SDS-PAGE gel showing protein content in ammonium sulphate fractions from *B. oleracea*; Extracts were subjected to ammonium sulphate precipitation as described in Methods. Samples were separated by SDS-PAGE and subsequently stained for protein. The position of Rubisco’s large (LS) and small (SS) subunits are indicated in the figure. 0.6 μg of sample was added per lane. **(D)** Western blot showing Rubisco content in ammonium sulphate fractions from *B. oleracea*; The relative intensities of the LS were calculated to be 0.83 ± 0.06 (CE), 0.85 ± 0.03 (8000 × g), 0.89 ± 0.06 (35% SN), 0.61 ± 0.12 (35% pell) and 1 (60% pell).

In order to test the suitability of HIC, lysed samples from both *S. oleracea* and *B. oleracea* were subjected to (NH_4_)_2_SO_4_ precipitation, with the majority of Rubisco protein precipitating between 35 and 60% saturation of (NH_4_)_2_SO_4_ (Figure 1). As expected, this observation is in agreement with previous reports where (NH_4_)_2_SO_4_ precipitation has been employed to extract Rubisco from higher plant species, including *S. oleracea* and *Arabidopsis*[24,32].

### HIC reveals the presence of hydrophobically distinct Rubisco fractions

**Figure 2:**
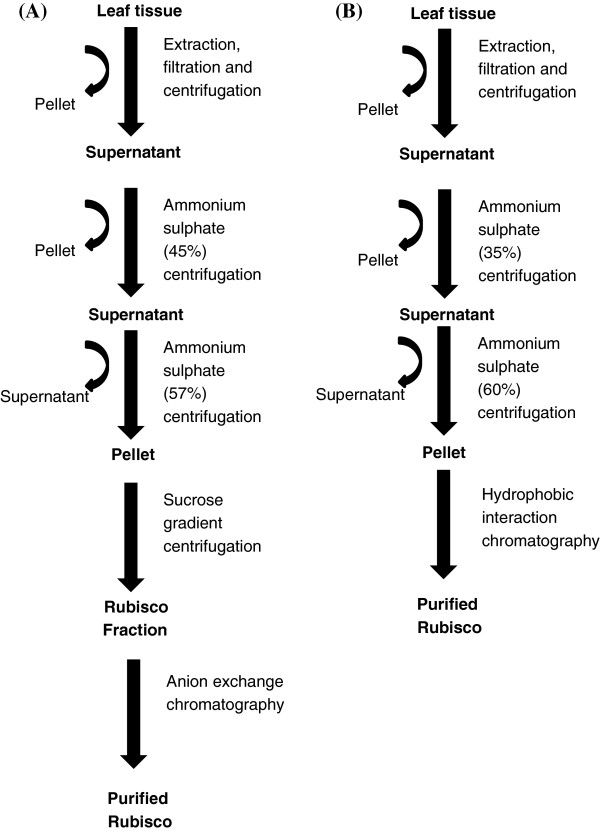
**Flow-chart outlining a comparison of HIC and IEC Rubisco purification protocols.** A comparison is made between **(A)** a commonly employed IEC protocol reported by Sulvucci *et al.*[24] and **(B)** the combined ammonium sulphate and HIC protocol reported in this paper.

**Figure 3:**
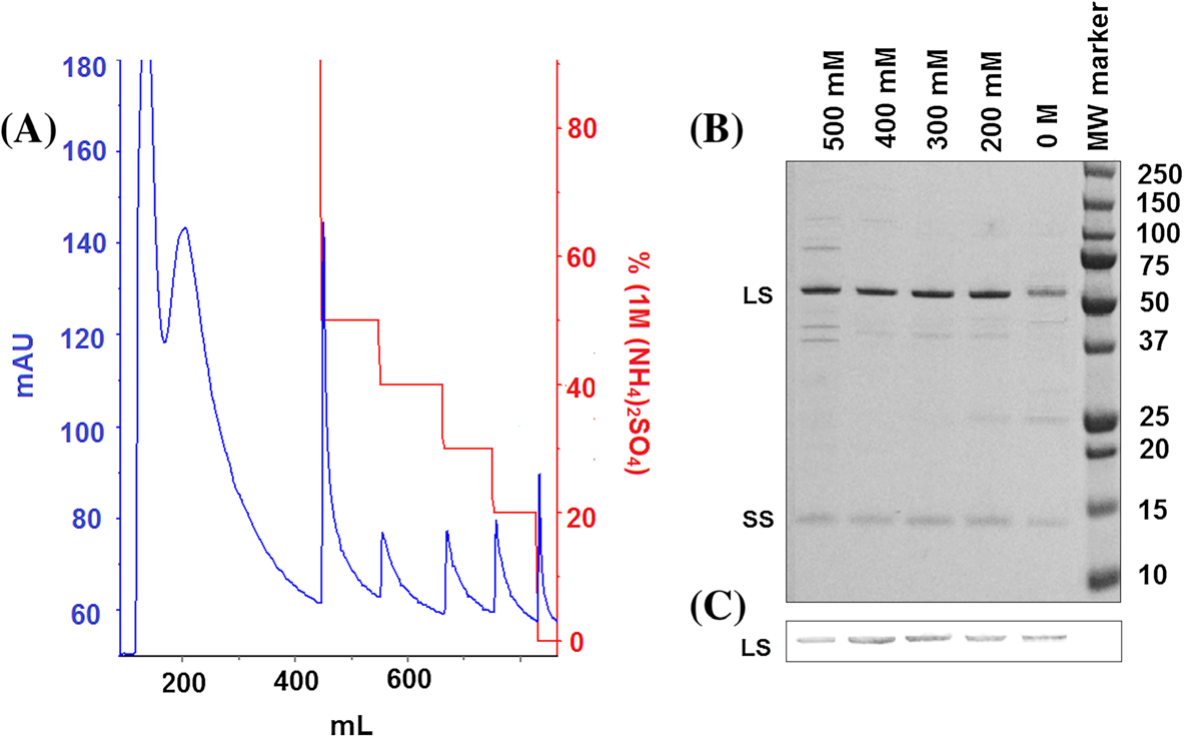
**HIC elution profile from the 60% ammonium sulphate pellet isolated from *S. oleracea*, and the SDS-PAGE gel and western blot showing protein content from the elution steps of the HIC purification. (A)** HIC elution profile generated by loading the 60% (NH_4_)_2_SO_4_ pellet isolated from *S. oleracea*; The ammonium sulphate pellet (60% pell; see Figure 1) was loaded onto a 5 mL HiTrap HIC column, washed and eluted as described in Methods. The gradient used for the separation (red line) and the positions of the major absorption peaks obtained by measuring OD at 280 nm (blue line) following the HIC elution profile is shown. Elutions were performed at 500 mM, 400 mM, 300 mM, 200 mM, 0 M ammonium sulphate. **(B)** SDS-PAGE gel showing protein content from the different steps involved in the HIC purification protocol of Rubisco isolated from *S. oleracea*; Samples were separated by SDS-PAGE and subsequently stained for protein. The position of Rubisco’s large (LS) and small (SS) subunits are indicated in the figure. 0.6 μg of sample was added per lane. The purity of the Rubisco fractions were calculated as 65.2 ± 4.7% (500 mM), 91.9 ± 3.1%, (400 mM), 92.6 ± 2.2%, (300 mM), 90.2 ± 2.4% (200 mM) and 76.6 ± 3.1% (0 M). **(C)** Western blot showing Rubisco content from HIC purified *S. oleracea* Rubisco fractions; The relative intensities of the LS for the four high purity Rubisco fractions were 0.97 ± 0.03, (400 mM), 0.98 ± 0.02, (300 mM), 0.94 ± 0.04 (200 mM) and 0.84 ± 0.06 (0 M). The gels and western blot figures are representative of 3 sample sets.

**Figure 4:**
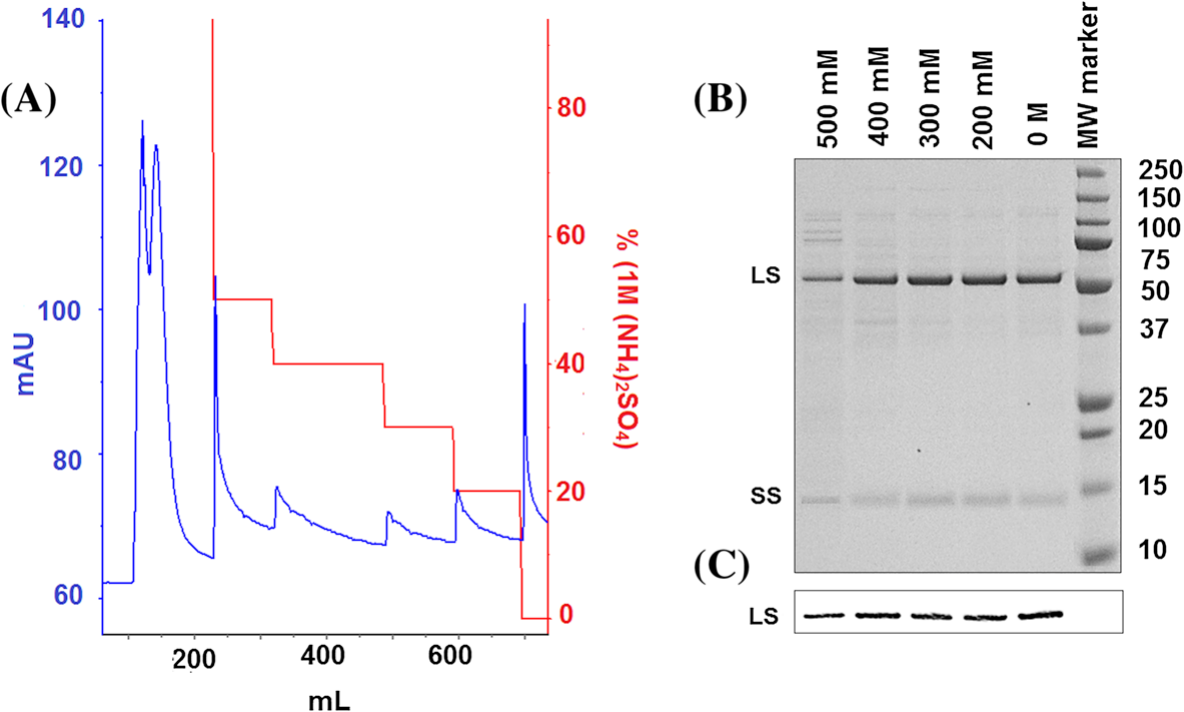
**HIC elution profile from the 60% ammonium sulphate pellet isolated from *B. oleracea*, and the SDS-PAGE gel and western blot showing protein content from the elution steps of the HIC purification. (A)** HIC elution profile generated by loading the 60% (NH_4_)_2_SO_4_ pellet isolated from *B. oleracea*; The ammonium sulphate pellet (60% pellet; see Figure 1) was loaded onto a 5 mL HiTrap HIC column, washed and eluted as described in Methods. The gradient used for the separation (red line) and the positions of the major absorption peaks obtained by measuring OD at 280 nm (blue line) following the HIC elution profile is shown. Elutions were performed at 500 mM, 400 mM, 300 mM, 200 mM, 0 M ammonium sulphate. **(B)** SDS-PAGE gel showing protein content from the different steps involved in the HIC purification protocol of Rubisco isolated from *B. oleracea*; Samples were separated by SDS-PAGE and subsequently stained for protein. The position of Rubisco’s large (LS) and small (SS) subunits are indicated in the figure. 0.6 μg of sample was added per lane. The purity of the Rubisco fractions were calculated as 59.2 ± 4.2% (500 mM), 82.3 ± 6.0% (400 mM), 92.8 ± 1.3% (300 mM), 96.1 ± 0.2% (200 mM) and 95.2 ± 2.7% (0 M). **(C)** Western blot showing Rubisco content from HIC purified *B. oleracea* Rubisco fractions; The relative intensities of the LS for the four high purity Rubisco fractions were calculated as 0.76 ± 0.11 (400 mM), 0.92 ± 0.07 (300 mM), 0.98 ± 0.02 (200 mM) and 0.97 ± 0.06 (0 M). The gels and western blot figures are representative of 3 sample sets.

As the HIC column binds proteins at high salt concentration, the (NH_4_)_2_SO_4_ fraction containing Rubisco was directly subjected to our HIC protocol (without the need to desalt the sample). Our method is summarised in Figure 2, which also shows a typical Rubisco isolation and purification protocol taken from Salvucci *et al*. for comparison [24]. Bound proteins were step-eluted in order to gain reproducibility, while conserving simplicity in handling (facilitating the potential use of syringe operated columns). A typical UV trace of the HIC elution profile demonstrates that a substantial proportion of the loaded proteins from *S. oleracea* (Figure 3A) and *B. oleracea* (Figure 4A), either did not bind to the column, or were eluted in the 1 M (NH_4_)_2_SO_4_ wash step or in the 500 mM step elution (see Additional file 1: Tables S1 and S2 for HIC purification tables). Interestingly there were five prominent peaks, observed at 500 mM, 400 mM, 300 mM, 200 mM and 0 M salt concentrations, all of which contained Rubisco (shown in the SDS-PAGE gel, Figures 3B and 4B, and confirmed by the presence of the large subunit in the western blot, Figures 3C and 4C), although it is of note that the 500 mM fractions had relatively low purities of 65% (*S. oleracea*) and 59% (*B. oleracea*) (calculated by analysis of SDS-PAGE gels, Figures 3B and 4B). The UV trace of these step elution fractions revealed the presence of relatively broad peaks and tails, supporting the use of step elution. For each concentration of ammonium sulphate used in the step elution of protein in our HIC protocol, the column was allowed to equilibrate, such that the UV absorbance returned to the baseline. This ensured that all protein at the corresponding salt concentration had been eluted prior to proceeding with the next step elution.

The 0 M Rubisco fraction isolated from *S. oleracea* had a purity of 77%, with the main impurities (as judged by densitometric scans of Coomassie stained gels, Figure 3B) being proteins with molecular weights of ~45 kDa and ~26 kDa. In contrast, the 400 mM, 300 mM and 200 mM Rubisco fractions (isolated from *S. oleracea*) showed relatively high purities (between 90-93%) with the main impurity present in all three fractions being a ~41 kDa protein. The 200 mM fraction contained an additional ~26 kDa protein.

Interestingly, the HIC fractions obtained from *B. oleracea* extracts revealed some species-specific hydrophobic differences. While extracts from both species could be separated into fractions at 400 mM, 300 mM, 200 mM and 0 M salt concentrations, differences in the purity of these fractions were observed when compared with the results from *S. oleracea*. For example, the results from *B. oleracea* showed that the 300 mM, 200 mM and 0 mM HIC fractions had high purity (93-96%), with the 300 mM fraction also containing a ~39 kDa protein impurity. Interestingly the fraction that eluted at 400 mM salt showed a lower purity of 82% (Figure 4B), with the main impurities being due to ~43 kDa and ~39 kDa proteins. This is in contrast to the results from *S. oleracea* where we observed the 0 M eluate to have a lower purity.

The protein impurities (41 kDa and 45 kDa for *S. oleracea*) in these HIC fractions are of comparable molecular weight to the smaller and larger isoforms of Rubisco activase [33,34], (a protein that has been indicated to associate with Rubisco [35]). Although there is no available data on the molecular weights of Rubisco activase isoforms from *B. oleracea*, it was speculated that the protein impurities (39 kDa and 43 kDa) in these HIC fractions could be due to the presence of the smaller and larger isoforms of Rubisco Activase. However, probing these fractions with antibodies against Rubisco activase did not support this notion in either species (data not shown).

Taken together, our data demonstrates the suitability of HIC as a method for obtaining high purity Rubisco. Rubisco extracted from the two species tested here (*S. oleracea* and *B. oleracea*) could be purified to similar levels to those reported using a rapid FPLC method, which obtained Rubisco from *S. oleracea* with 93% purity [20]. It is worth noting that no significant degradation products of the large Rubisco subunit could be observed in the HIC fractions (Figures 3C and 4C), indicating that Rubisco was not subject to proteolytic damage throughout the purification described here. This corroborates the protein integrity preserving character of the HIC method.

To ensure that the hydrophobically distinct populations of Rubisco were not a result of the use of (NH_4_)_2_SO_4_ during the precipitation step or during HIC, crude extracts from *S. oleracea* were loaded straight onto the HIC column (omitting the (NH_4_)_2_SO_4_ precipitation step), followed by elution with potassium chloride (KCl) instead of (NH_4_)_2_SO_4_. We again observed hydrophobically distinct fractions of Rubisco under these conditions, using this HIC protocol, suggesting that the different fractions of Rubisco are not a result of the use of (NH_4_)_2_SO_4_ (Additional file 2).

At this stage, we can only speculate as to why we observe hydrophobically distinct populations of Rubisco in both species. Separation by HIC clearly implies that the Rubisco populations differ in their hydrophobicity, at least on the surface of the protein. As Rubisco is a hugely complex enzyme, HIC could potentially be separating Rubisco populations where one or more of the enzyme’s eight subunits might have undergone a conformational change, with the HIC fractions capturing the resulting populations generated through subunit heterogeneity. Alternatively, the difference in hydrophobicity could be due to protein – protein interactions (e.g. potentially with one of the impurities found in these fractions) or post-translational modifications. Furthermore, it is possible that the distinct Rubisco fractions are capturing diverse holoenzyme populations of Rubisco, with differing combinations of small subunits. As the small subunits provide structural stability [8], any difference in small subunit combinations could affect Rubisco’s hydrophobic character. The distinct populations, and the protocol to obtain them, could therefore lead to exciting developments in understanding structural and interactome studies of this vital enzyme that will benefit current and future photosynthetic researchers.

### Kinetic analysis of the HIC fractions from *S. oleracea* and *B. oleracea* extracts

**Figure 5:**
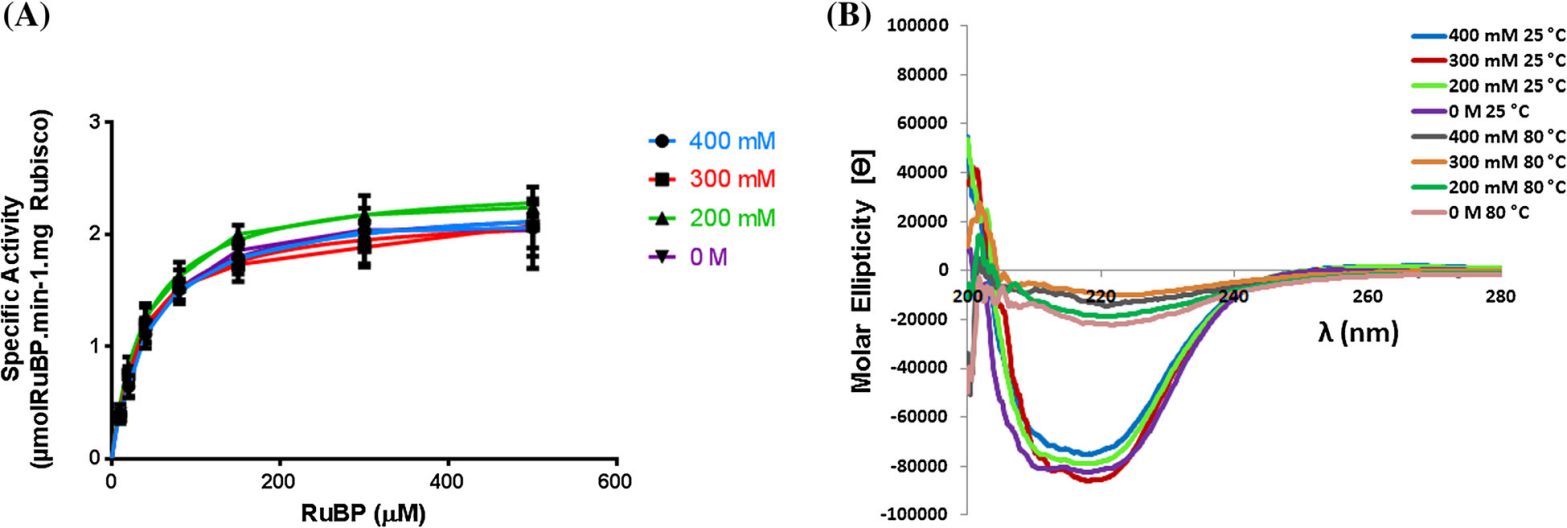
**Kinetic and structural characterisation of four HIC purified Rubisco fractions isolated from *B. oleracea *extracts. (A)** Determination of the K_M_ and V_MAX_ values for the substrate RuBP for *S. oleracea* HIC purified fractions; Average V_MAX_ values obtained for the 400, 300, 200 and 0 mM HIC fractions were 2.31 ± 0.16, 2.20 ± 0.11, 2.35 ± 0.07 and 2.23 ± 0.07 μmol.min^-1^.mgRubisco^-1^, respectively. Average K_M_ values obtained were 44 ± 11, 39 ± 7, 39 ± 4 and 45 ± 6 μM respectively. Kinetic calculations and curve-fitting was done using GraphPad Prism 6 software. Error bars shown are standard deviation with n = 6 (3 different biological replicates measured in duplicate). **(B)** Circular dichroism spectra of *S. oleracea* Rubisco fractions purified using HIC; The 400 mM, 300 mM, 200 mM and 0 M HIC fractions were was run at 25°C and then at 80°C, at concentration between 0.15-0.25 mg/mL. Data are shown as an average molar ellipticity [θ] of 3 biological repeats.

**Figure 6:**
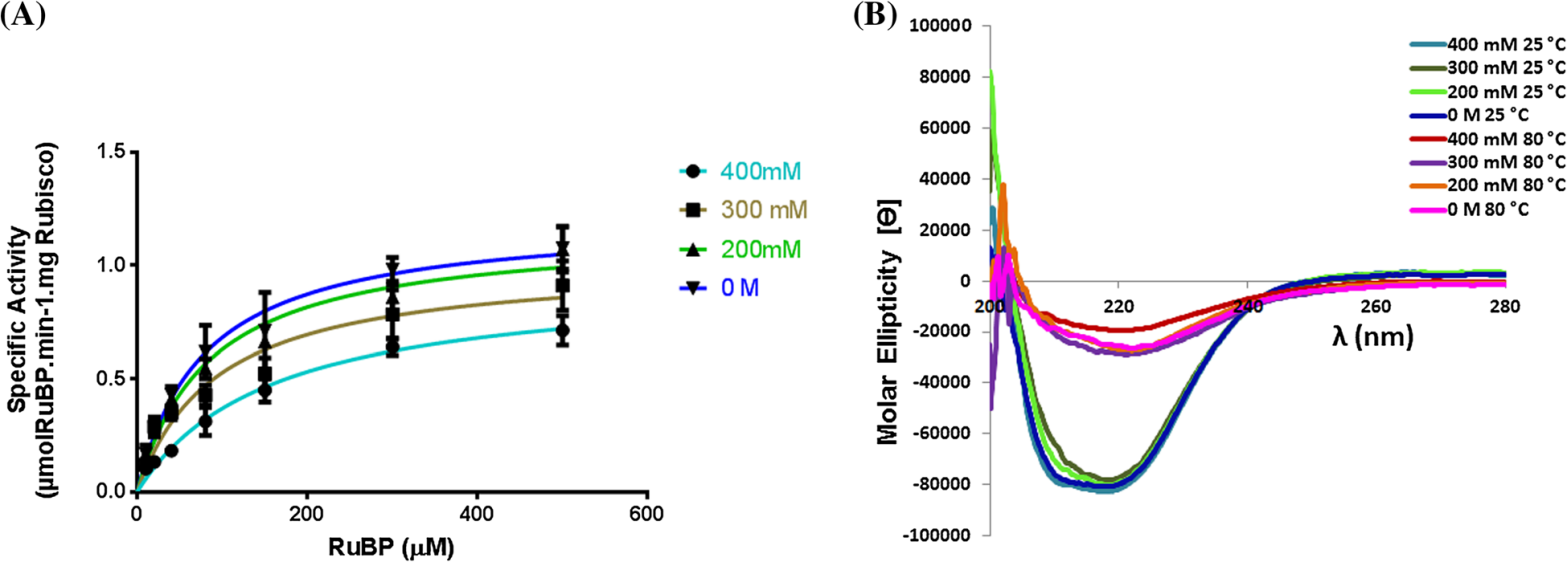
**Kinetic and structural characterisation of four HIC purified Rubisco fractions isolated from *S. oleracea *extracts. (A)** Determination of the K_M_ and V_MAX_ values for the substrate RuBP for *B. oleracea* HIC purified fractions; Average V_MAX_ values obtained for the 400, 300, 200 and 0 mM HIC fractions were 0.94 ± 0.08, 1.02 ± 0.11, 1.16 ± 0.09 and 1.21 ± 0.10 μmol.min^-1^.mgRubisco^-1^ respectively. Average K_M_ values obtained were 147 ± 35, 92 ± 29, 83 ± 19 and 77 ± 19 μM respectively. Kinetic calculations and curve-fitting was done using GraphPad Prism 6 software. Error bars shown are standard deviation with n = 6 (3 different biological replicates measured in duplicate). **(B)** Circular dichroism spectra of *B. oleracea*. Rubisco fractions purified using HIC; The 400 mM, 300 mM, 200 mM and 0 M Rubisco fractions were was run at 25°C and then at 80°C, at concentration between 0.15-0.25 mg/mL. Data are shown as an average molar ellipticity [θ] of 3 biological repeats.

The observed elution profiles from *S. oleracea* and *B. oleracea* extracts imply that there are differences in the hydrophobic character of Rubisco eluted in these different fractions. To probe whether the difference in the hydrophobic character could influence Rubisco’s enzymological properties, we determined the K_M_ and K_CAT_ values for all Rubisco containing fractions. The Michaelis-Menten plot reveals that all the different fractions containing Rubisco, isolated using HIC, from *S. oleracea* (Figure 5A) have similar enzymological properties. The Michaelis-Menten plot reveals that the enzymological properties of the 300 mM, 200 mM and 0 M HIC Rubisco containing fractions isolated from *B. oleracea* are also similar (Figure 6A). In contrast, the Rubisco activity in the 400 mM HIC fraction from *B. oleracea* extracts has a significant lower K_CAT_ and a higher K_M_ as compared to the 200 mM and 0 M HIC fractions.

The K_CAT_ and K_M_ values for Rubisco from *S. oleracea* (see Table 1) are in reasonable agreement with previously published data [20,31]. As enzymological studies on Rubisco extracted from *B. oleracea* have not been previously published, we report here for the first time the K_CAT_ for *B. oleracea* Rubisco (for the 300, 200 and 0 mM HIC fractions), and note that although a factor of 2 lower than *S. oleracea*, it is in reasonable agreement with values obtained from other C3 species (Table 1) [31]. The K_M_ (RuBP) values (for the 300, 200 and 0 mM HIC fractions) for *B. oleracea* (Table 1) are 2–3 times higher than that for *S. oleracea*, but are again in reasonable agreement to C3 species reported by Yeoh *et al.*[36].

**Table 1:** **Summary of V**_**MAX, **_**K**_**CAT **_**and K**_**M **_**values of the HIC purified Rubisco fraction from *S. oleracea *** **and *B. oleracea *** **extracts**

**Species**	**[(NH**_**4**_**)**_**2**_**SO**_**4**_**] mM**	**VMAX (μmol.min**^**-1**^**.mgRubisco**^**-1**^**)**	**K**_CAT_^* ^**(s**^**-1**^**)**	**KM (RuBP) (μM)**
*S. oleracea*	400	2.31 ± 0.16	2.63 ± 0.20	44 ± 11
	300	2.20 ± 0.11	2.52 ± 0.14	39 ± 7
	200	2.35 ± 0.07	2.76 ± 0.10	39 ± 4
	0	2.23 ± 0.06	2.59 ± 0.08	45 ± 6
*B. oleracea*	400	0.94 ± 0.08	1.09 ± 0.09	147 ± 35
	300	1.02 ± 0.11	1.17 ± 0.13	92 ± 29
	200	1.16 ± 0.09	1.34 ± 0.10	83 ± 19
	0	1.21 ± 0.10	1.39 ± 0.11	77 ± 19

*K_CAT_ was calculated based on 8 active sites and a molecular weight of 550 K g/mol.

Since there is a significant difference in sequence homology between Rubisco from *S. oleracea* and *B. oleracea* (71.7% for the small subunits of *S. oleracea* (RBCS2), and *B. oleracea* (BRA034024) and 91.4% for the large subunits), it is not unexpected that we should observe species specific differences in the HIC elution profiles and enzymological kinetics of Rubisco. However, the presence of distinct hydrophobic populations of Rubisco in both *S. oleracea* and *B. oleracea* suggests this phenomenon is not species specific.

An additional experiment was designed to investigate whether HIC could separate distinct subpopulations of Rubisco from Rubisco previously purified using IEC. Extracts from *S. oleracea* were purified by IEC and the Rubisco containing IEC fraction was then loaded onto an HIC column. Hydrophobically distinct fractions of Rubisco were again observed when eluted from the HIC column (Additional file 3). This further highlights the significance and potential use of this newly developed HIC protocol for Rubisco purification, and demonstrates the potential use of HIC in conjunction with the well-established IEC protocols.

### Circular dichroism studies reveal no significant structural differences in the Rubisco populations

Circular dichroism (CD) can be a useful spectroscopic method to study conformational changes of proteins [37-39]. CD studies were carried out on the four HIC fractions derived from both *S. oleracea* and *B. oleracea* extracts, to probe the cause of the differences in the hydrophobicity of the samples (Figures 5B and 6B respectively). For comparison, an IEC fraction of the corresponding extracts was also analysed (Additional file 4). Interestingly, each of the hydrophobically distinct fractions of Rubisco had similar values of molar ellipticites [θ] (~ -8000 deg cm^2^ dmol^-1^) and structures of the CD spectra when compared to each other, to the IEC fraction, and with CD spectra of Rubisco previously reported in the literature [40,41]. For comparison, maximum denaturation of the fractions was achieved by heating at 80°C (shown in Figures 5B and 6B), which caused a large drop in the molar ellipticities of the samples (≤2000 deg cm^2^ dmol^-1^), as well as a loss of structure in the spectra. This analysis demonstrates that there are no significant changes in the secondary structures of the Rubisco populations. Fractions purified using HIC or IEC showed similar CD spectra, suggesting that the Rubisco in the HIC fractions has the same structural integrity as the IEC purified Rubisco. With the latter being currently the standard method for obtaining pure Rubisco [20], the HIC method investigated here seems to produce pure Rubisco of comparable nature and quality. Furthermore, the CD experiments confirm that the heterogeneity of the Rubisco populations is not due to denaturation.

## Conclusions

Taking these findings together, we can conclude that HIC is not only a valuable alternative purification method for form I Rubisco, but it also has the unique capability of being able to resolve distinct Rubisco populations. Since Rubisco isolated using HIC has similar values of purity and catalytic activity to that obtained using existing IEC purification methods, HIC has the potential to replace IEC, providing the additional benefit of avoiding high dilutions, dialysis and gel filtration chromatography. In addition, the ability to separate distinct Rubisco populations makes HIC a valuable method for current and future Rubisco researchers. This is of particular importance for investigators seeking to determine Rubisco’s structure and interactome, since conformational alterations could significantly impact upon these results.

## Methods

### Chemicals and equipment

Chemicals for SDS-PAGE were obtained from Invitrogen. Instant Blue (Expedion) was used as a Coomassie staining agent. All other chemicals and enzymes were obtained from Sigma, except phosphoglycerate kinase (PGK) which was expressed and purified (see below). For homogenization of plant material, a Kenwood blender was used. Centrifugation was carried out in a Biofuge Primo R (Thermo Scientific). Purification was performed using an AKTA™ design FPLC system. SDS-PAGE gels and western blots were documented using a Fujifilm LAS-3000 Imaging System. The purity of the gels were determined using Image J software. Rubisco activity assays were performed in a Thermo Scientific Varioskan Flash Multimode reader.

### Extraction of Rubisco from leaves

Unless stated otherwise, all procedures were performed rapidly at 4°C to maximize active enzyme recovery. The mid ribs of *Spinach* (*S. oleracea*) or *Savoy Cabbage* (*B. oleracea)* leaves were removed and the plant tissue was quickly frozen in liquid nitrogen and powdered in a mortar and pestle. 75 mL of extraction buffer (20 mM Hepes (pH 6.5) 5 mM MgCl_2_, 0.33 M sorbitol, 0.2% (w/v) iso-ascorbic acid, 5 mM DDT, 0.75 mL of plant protease inhibitor cocktail (Sigma)) was added to 15 g of plant tissue, and homogenization and cell lysis carried out in a blender using 20–30 pulses, of approximately 1–2 second duration. The homogenate was filtered through two layers of Miracloth (Calbiochem) followed by centrifugation for 30 min at 3000 × g to remove intact cells and debris, and the cell extract (CE) was further centrifuged for 10 min at 8000 × g. The supernatant was subjected to (NH_4_)_2_SO_4_ precipitation.

### Ammonium sulphate precipitation

Two rounds of (NH_4_)_2_SO_4_ precipitation were performed at 35 and 60% saturation. In each case, solid (NH_4_)_2_SO_4_ was added to the desired percentage of saturation and the solution was stirred and left to equilibrate for 20 min. The precipitate was collected by centrifugation for 10 min at 10000 × g. The supernatant of the 35% saturation was subjected to the next round of precipitation. Protein pellets may be stored at 4°C for 2 days.

### Hydrophobic interaction chromatography using ammonium sulphate

Purification was performed with a flow rate of 4 mL/min using a 5 mL HiTrap™ Phenyl Sepharose 6 FF (high sub) column (GE Healthcare). Buffers were filtered before use through 0.45 μm filters. (NH_4_)_2_SO_4_ pellets were resuspended in HIC buffer (50 mM Tris–HCl (pH 7.6, KOH), 20 mM MgCl_2_, 20 mM NaHCO_3_, 0.2 mM EDTA, 2 mM DTT) containing 1 M (NH_4_)_2_SO_4_, equilibrated and filtered through a 0.45 μm filter to prevent clogging of the column. After washing the HIC column with 25 mL HIC buffer, the column was equilibrated with 25 mL HIC buffer (containing 1 M (NH_4_)_2_SO_4_), and then the sample was loaded. After washing the column with HIC buffer (containing 1 M (NH_4_)_2_SO_4_), a series of step elutions were performed at (NH_4_)_2_SO_4_ concentrations of 500, 400, 300, 200 and 0 mM (the column was washed between each elution step until the UV absorbance returned to baseline). Elution peaks were collected and desalted using 30 kDa filter units (washed 4 times with HIC buffer at 5000 × g for 10 mins), and the subsequent samples were analysed by SDS-PAGE, Western blot, and for Rubisco activity (for protocols, see below). Protein concentrations were determined by Bradford assay [42].

### Hydrophobic interaction chromatography using potassium chloride

Purification was performed with a flow rate of 4 mL/min using a 5 mL HiTrap™ Phenyl Sepharose 6 FF (high sub) column (GE Healthcare). Buffers were filtered before use through 0.45 μm filters. Crude extract samples were filtered through a 0.45 μm filter to prevent clogging of the column. After washing the HIC column with 25 mL HIC buffer, the column was equilibrated with 25 mL HIC (containing 2 M KCl), and then the sample was loaded. After washing the column with HIC buffer containing 2 M KCl, a series of step elutions were performed at KCl concentrations of 1.5 M, 1.0 M, 0.6 M, 0.3 M and 0 M (the column was washed between each elution step until the UV absorbance returned to baseline). Elution peaks were collected and desalted using 30 kDa filter units (washed 4 times with HIC buffer at 5000 × g for 10 mins), and analysed for Rubisco activity. Protein concentrations were determined by Bradford assay.

### Ion exchange chromatography followed by hydrophobic interaction chromatography

Purification was performed with a flow rate of 3 mL/min using 3 × 5 mL HiTrap™ Q HP columns for increased yield (GE Healthcare). Buffers were filtered before use through 0.45 μm filters. (NH_4_)_2_SO_4_ pellets were resuspended in IEC buffer (25 mM Tris–HCl (pH 7.6, KOH), 10 mM MgCl_2_, 10 mM NaHCO_3_, 0.1 mM EDTA, 2 mM DTT) and filtered through a 0.45 μm filter to prevent clogging of the column. After washing the IEC column with 25 mL IEC buffer containing 1 M KCl, the column was equilibrated with 25 mL of IEC buffer, and then the sample was loaded. After washing the column with IEC buffer, the sample was eluted over a linear gradient from 0 to 0.5 M KCl, over a 90 minute period. Rubisco is known to elute between ~ 0.30-0.35 M KCl.

The Rubisco peak was collected and desalted using 30 kDa filter units (washed 4 times with HIC buffer at 5000 × g for 10 mins), and analysed for Rubisco activity. The remaining sample was made up to 1 M (NH_4_)_2_SO_4_ in HIC buffer, and filtered through a 0.45 μm filter. After washing the HIC column (5 mL HiTrap™ Phenyl Sepharose 6 FF (high sub)) with 25 mL HIC buffer, the column was equilibrated with 25 mL HIC (containing 1 M (NH_4_)_2_SO_4_), and then the sample was loaded. After washing with HIC buffer containing 1 M (NH_4_)_2_SO_4_, a series of step elutions were performed at (NH_4_)_2_SO_4_ concentrations of 500, 400, 300, 200 and 0 mM (the column was washed between each elution step until the UV absorbance returned to baseline). Elution peaks were collected and desalted using 30 kDa filter units (washed 4 times with HIC buffer at 5000 × g for 10 mins), and analysed for Rubisco activity. Protein concentrations were determined by Bradford assay.

### SDS polyacrylamide gel electrophoresis (SDS-PAGE)

SDS-PAGE was performed in NuPage® Bis-Tris 4-12% gradient gels (Invitrogen) according to the manufacturer’s protocol [43]. Staining was carried with InstantBlue Coomassie stain.

### Western blot

After SDS-PAGE, the gel was equilibrated in 4°C Transfer buffer (Tris 25 mM, Glycine 192 mM, Methanol reagent grade 20% (v/v)) for 15 mins. Immunoblotting using a Criterion™ Blotter Cell (Bio-rad) was carried out at 70 V for 90 minutes using a Bio-rad Powerpac® 1000. After blotting, the nitrocellulose membrane was washed twice in TBST, pH 7.5 (Tris 50 mM, NaCl 150 mM , Tween 20, 0.05% (v/v)) at room temperature on an orbital shaker, and followed by washing with Blocking solution (2% (w/v) milk powder dissolved in TBST) for 2 hours at room temperature. The membrane was incubated with primary Antibody Solution (rabbit anti-RbcL (Agrisera) dissolved in 0.5% Blocking Solution at 1:5000) overnight at 4°C, followed by 5 washes with TBST, for 3 minutes each. The membrane was incubated with secondary Antibody Solution (donkey anti-rabbit IgG (H&L) HRP conjugated (Agrisera) dissolved in 0.5% Blocking Solution at 1:10000) for 2 hours, after which, the membrane was washed 3 times at 10 minute intervals with TBST. Lumi-Light substrate (Roche) was mixed with the membrane for 5 minutes and then the membrane was quickly imaged.

### Rubisco activity assay

Rubisco carboxylase activity was determined using a non-radioactive microplate-based assay, which determines the product [(3-phosphoglycerate (3-PGA)] in an enzymic cycle between glycerol-3-phospahte dehydrogenase and glycerol-3-phospate oxidase, adapted from Sulpice *et al*. [44]. The assay was monitored through the oxidation of NADH by optical density measurements at 340 nm at 25°C. 15 μL of Rubisco was added to the initial buffer for activation (containing 100 mM Tricine (pH 8.0, KOH), 20 mM MgCl_2_, 2 mM EDTA and 10 mM NaHCO_3_), to give a total volume of 30 μL. The concentration of NaHCO_3_ was confirmed to be saturating. While incubating the sample for 15 mins at room temperature, 15 uL of different concentrations of RuBP (giving final concentrations of 10 μM to 500 μM) was pipetted into a 96 well plate. The Rubisco/initial buffer mixture was then added to the wells (giving a total volume of 45 μL) and allowed to react for 60 seconds, after which, 15 μL of absolute ethanol was added to stop the reaction. To minimise the amount of assay enzymes used, 20 μL of the assay mixture was added to a 384 well plate (after 5 mins of reaction with ethanol), and 20 μL of determination buffer was added to start the reaction [final concentrations in 40 μL were: 1.875 u/mL phosphoglycerate kinase (PGK), 3 u/mL glyceraldehyde-3-phosphate dehydrogenase (GAPDH), 2.5 u/mL α-glycerol-3-phosphate dehydrogenase-/triose-P isomerase (G3PDH-TPI), 100 u/mL glycerol-3-phosphate oxidase (G3P-OX), 700 u/mL catalase, 3 mM ATP, 0.5 mM NADH, 1 mM MgCl_2_, 60 mM Tricine (pH 8.0, KOH)]. The rates of reaction were calculated as the decrease of the absorbance in OD.min^-1^ and converted to μmol by use of a 3-phosphoglyceric acid (3PGA) calibration curve.

### Expression and purification of phosphoglycerate kinase

The coding region of the DNA sequence of phosphoglycerate kinase 1 **(***Saccharomyces cerevisiae* ***)*** was cloned into pGEX-6P-1 expression vector (GE Healthcare). Phosphoglycerate kinase 1 expression and purification was performed as previously described [45].

### Circular dichroism

CD spectra were measured using a JASCO-715 Circular Dichroism Spectropolarimeter, with the use of using JASCO PTS-604 T Temperature Controller, to regulate the temperature. Samples were allowed to incubate at 25°C and 80°C for 10 minutes before CD measurements were taken. Molecular ellipticities [θ] are reported, using an average molecular weight of 560 KDa for Rubisco, and a path length of 0.1 cm. Sample were prepared in HIC buffer at protein concentrations between 0.15-0.25 mg/mL.

## Abbreviations

HIC: Hydrophobic interaction chromatography; IEC: Ion exchange chromatography; RuBP: Ribulose-1,5-bisphophate; RLPs: Rubisco like proteins; CA1P: 2 carboxy-D-arabinitol 1-phosphate; SDS-PAGE: Sodium dodecyl sulphate polyacrylamide gel electrophoresis; CD: Circular dichroism; PGK: Phosphoglycerate kinase; CE: Cell extract; 3PGA: 3-phosphoglyceric acid; GAPDH: Glyceraldehyde-3-phosphate dehydrogenase; G3PDH-TPI: mL α-glycerol-3-phosphate dehydrogenase-/triose-P isomerase; G3POX: Glycerol-3-phosphate oxidase; IPTG: Isopropyl β-D-1-thiogalactopyranoside; GST: Glutathione S-transferase.

## Competing interests

The authors declare that they have no competing interests.

## Authors’ contributions

KOD carried out the purification study, enzymological studies, and CD analysis. PP and ZG assisted in the purification and enzymological studies. MSD and SK carried out preliminary purification studies with *B. oleracea*. LHM expressed and purified phosphoglycerate kinase. RW and LB conceived the study and participated in its design. KOD, RW and LB coordinated and drafted the manuscript. All authors read and approved the final manuscript.

## Supplementary Material

Additional file 1 **Purification tables for *S. oleracea *** **(Table S1) and *B. oleracea *** **(Table S2).** NB. * not calculated for these samples. ^-^ Activity was too low to measure. ^a^Calculations based on ‘cell extract’. ^b^Calculations based on ‘load’. ^#^ K_CAT_ calculated based on 8 active sites of Rubisco, with a total molecular weight of 550 K g/mol. Click here for file

Additional file 2 **HIC elution profile (using potassium chloride (KCl)) from cell extract of *S. oleracea*; and the SDS-PAGE gel showing protein content from the different elution steps of the HIC purification protocol. (A)** HIC (using potassium chloride (KCl)) elution profile generated by loading cell extract isolated from S. oleracea;The cell extract was loaded at 2 M KCl onto a 5 mL HiTrap HIC column, washed and eluted as described in Methods**.** The gradient used for the separation (red line) and the positions of the major absorption peaks obtained by measuring OD at 280 nm (blue line) following the HIC elution profile is shown. Elutions were performed at 1.5 M, 1.0 M, 0.6 M, 0.3 M and 0 M KCl. **(B)** SDS-PAGE gel showing protein content from the different steps involved in the HIC potassium chloride purification protocol of Rubisco isolated from *S. oleracea*; Samples were separated by SDS-PAGE and subsequently stained for protein. The position of Rubisco’s large (LS) and small (SS) subunits are indicated in the figure. 0.4 μg of sample was added per lane. The purity of the CE sample was calculated as 68.2 ± 3.1%, and HIC fraction containing Rubisco had purities of 86.4 ± 1.4 (1.5 M), 87.7 ± 2.1% (1.0 M), 89.1 ± 1.7% (0.6 M), 81.3 ± 3.0% (0.3 M) and 70.1 ± 4.2% (0 M). The gels are representative of 2 sample sets. Click here for file

Additional file 3 **Kinetic characterisation of the IEC and HIC purified Rubisco fractions from *S. oleracea *** **extracts.** Determination of the K_M_ and V_MAX_ values for the substrate RuBP for *S. oleracea* Rubisco purified using IEC and followed by further purification of the IEC fraction using HIC; Rubisco was purified using IEC, and the subsequent fraction was then loaded onto an HIC column at 1 M (NH_4_)_2_SO_4_, and eluted at (NH_4_)_2_SO_4_ concentrations of 500 mM, 400 mM, 300 mM, 200 mM and 0 M, as per Methods section. Average V_MAX_ and K_M_ values obtained for the IEC fraction was 2.8 ± 0.22 μmol.min.mg and 47 ± 13 μM. Average V_MAX_ values obtained for, 300 mM, 200 mM and 0 M was 3.0 ± 0.16, 3.1 ± 0.10 and 2.8 ± 0.13 μmol.min.mg respectively. Average K_M_ values obtained were 46 ± 8, 40 ± 5 and 47 ± 8 μM respectively. It is of note that although there was a peak in the UV trace of the elution profile at 400 mM (NH_4_)_2_SO_4_ elution, there was not enough protein obtained to perform kinetic analysis, as the peak was very small (even when more protein was loaded). Kinetic calculations and curve-fitting was done using GraphPad Prism 6 software. Error bars shown are standard deviation with n = 6 (3 different biological replicates measured in duplicate). Click here for file

Additional file 4 **Circular dichroism spectra of *S. oleracea *** **Rubisco, purified using IEC.** Rubisco purified using IEC was run at 25°C at a concentration of 0.2 mg/mL. Data are shown as an average molar ellipticity [θ] of 3 biological repeats. Click here for file
