# Supplementary material for: Isolation and kinetic characterisation of hydrophobically distinct populations of form I Rubisco
Source: Plant Methods. 2014 Jun 12;10:17. doi: 10.1186/1746-4811-10-17 (PMC4076768; doi:10.1186/1746-4811-10-17)
Supplement: Additional file 1 — Purification tables for S. oleracea (Table S1) and B. oleracea (Table S2). NB. * not calculated for these samples. - Activity was too low to measure. aCalculations based on ‘cell extract’. bCalculations based on ‘load’. # KCAT calculated based on 8 active sites of Rubisco, with a total molecular weight of 550 K g/mol. [file 1746-4811-10-17-S1.docx]

**Table S1**: Purification table for *S. oleracea*

**Table S2**: Purification table for *B. oleracea*
