# Supplementary figures and images for: Isolation and kinetic characterisation of hydrophobically distinct populations of form I Rubisco
Source: Plant Methods. 2014 Jun 12;10:17. doi: 10.1186/1746-4811-10-17 (PMC4076768; doi:10.1186/1746-4811-10-17)

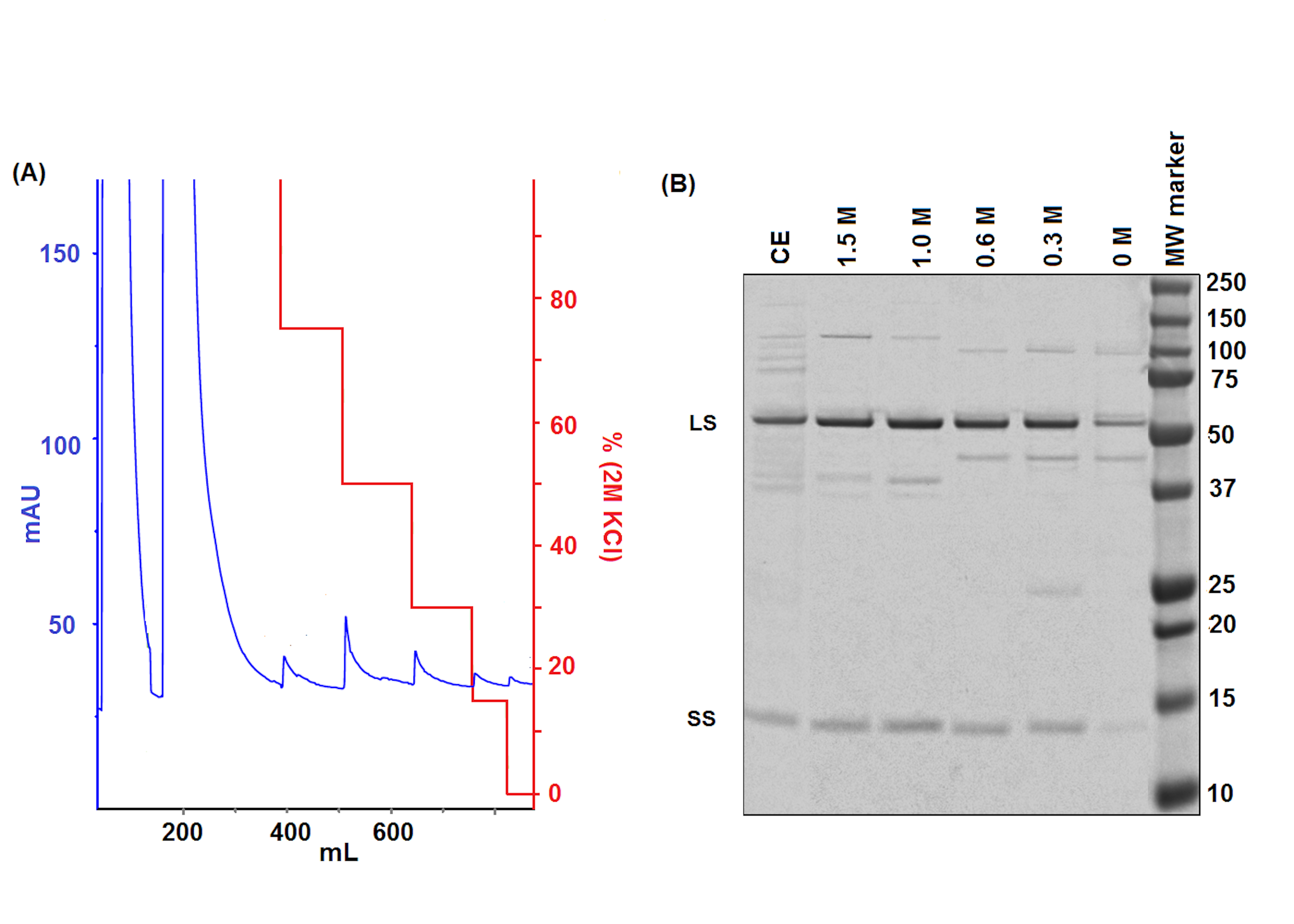

Supplement: Additional file 2 — HIC elution profile (using potassium chloride (KCl)) from cell extract of S. oleracea ; and the SDS-PAGE gel showing protein content from the different elution steps of the HIC purification protocol. (A) HIC (using potassium chloride (KCl)) elution profile generated by loading cell extract isolated from S. oleracea;The cell extract was loaded at 2 M KCl onto a 5 mL HiTrap HIC column, washed and eluted as described in Methods. The gradient used for the separation (red line) and the positions of the major absorption peaks obtained by measuring OD at 280 nm (blue line) following the HIC elution profile is shown. Elutions were performed at 1.5 M, 1.0 M, 0.6 M, 0.3 M and 0 M KCl. (B) SDS-PAGE gel showing protein content from the different steps involved in the HIC potassium chloride purification protocol of Rubisco isolated from S. oleracea; Samples were separated by SDS-PAGE and subsequently stained for protein. The position of Rubisco’s large (LS) and small (SS) subunits are indicated in the figure. 0.4 μg of sample was added per lane. The purity of the CE sample was calculated as 68.2 ± 3.1%, and HIC fraction containing Rubisco had purities of 86.4 ± 1.4 (1.5 M), 87.7 ± 2.1% (1.0 M), 89.1 ± 1.7% (0.6 M), 81.3 ± 3.0% (0.3 M) and 70.1 ± 4.2% (0 M). The gels are representative of 2 sample sets. [file 1746-4811-10-17-S2.png]

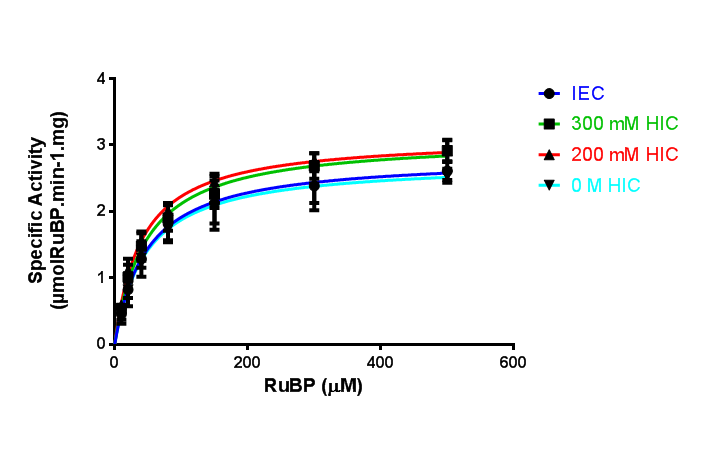

Supplement: Additional file 3 — Kinetic characterisation of the IEC and HIC purified Rubisco fractions from S. oleracea extracts. Determination of the KM and VMAX values for the substrate RuBP for S. oleracea Rubisco purified using IEC and followed by further purification of the IEC fraction using HIC; Rubisco was purified using IEC, and the subsequent fraction was then loaded onto an HIC column at 1 M (NH4)2SO4, and eluted at (NH4)2SO4 concentrations of 500 mM, 400 mM, 300 mM, 200 mM and 0 M, as per Methods section. Average VMAX and KM values obtained for the IEC fraction was 2.8 ± 0.22 μmol.min.mg and 47 ± 13 μM. Average VMAX values obtained for, 300 mM, 200 mM and 0 M was 3.0 ± 0.16, 3.1 ± 0.10 and 2.8 ± 0.13 μmol.min.mg respectively. Average KM values obtained were 46 ± 8, 40 ± 5 and 47 ± 8 μM respectively. It is of note that although there was a peak in the UV trace of the elution profile at 400 mM (NH4)2SO4 elution, there was not enough protein obtained to perform kinetic analysis, as the peak was very small (even when more protein was loaded). Kinetic calculations and curve-fitting was done using GraphPad Prism 6 software. Error bars shown are standard deviation with n = 6 (3 different biological replicates measured in duplicate). [file 1746-4811-10-17-S3.png]

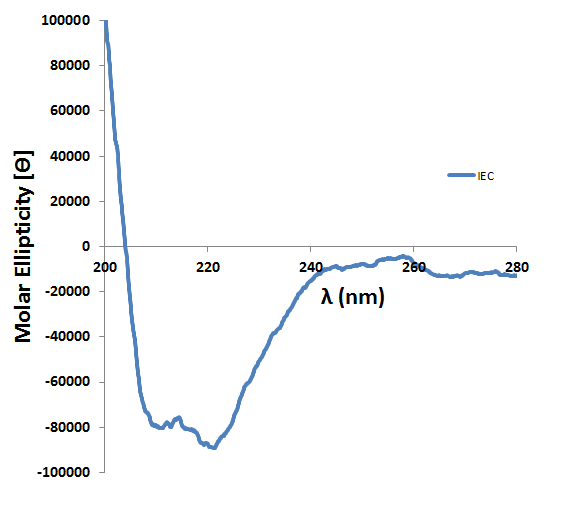

Supplement: Additional file 4 — Circular dichroism spectra of S. oleracea Rubisco, purified using IEC. Rubisco purified using IEC was run at 25°C at a concentration of 0.2 mg/mL. Data are shown as an average molar ellipticity [θ] of 3 biological repeats. [file 1746-4811-10-17-S4.png]
